# Suppression of inner blood-retinal barrier breakdown and pathogenic Müller glia activation in ischemia retinopathy by myeloid cell depletion

**DOI:** 10.1186/s12974-024-03190-9

**Published:** 2024-08-24

**Authors:** Lingli Zhou, Zhenhua Xu, Haining Lu, Hongkwan Cho, Yangyiran Xie, Grace Lee, Kaoru Ri, Elia J. Duh

**Affiliations:** 1 State Key Laboratory of Ophthalmology, Zhongshan Ophthalmic Center, Guangdong Provincial Key Laboratory of Ophthalmology and Visual Science, Sun Yat-Sen University, Guangzhou, China; 2 Wilmer Eye Institute, Johns Hopkins University School of Medicine, Baltimore, MD USA

**Keywords:** Microglia, Macrophages, Csf1r, Müller glia, Blood-retinal barrier, Neuroinflammation

## Abstract

Ischemic retinopathies including diabetic retinopathy are major causes of vision loss. Inner blood-retinal barrier (BRB) breakdown with retinal vascular hyperpermeability results in macular edema. Although dysfunction of the neurovascular unit including neurons, glia, and vascular cells is now understood to underlie this process, there is a need for fuller elucidation of the underlying events in BRB dysfunction in ischemic disease, including a systematic analysis of myeloid cells and exploration of cellular cross-talk. We used an approach for microglia depletion with the CSF-1R inhibitor PLX5622 (PLX) in the retinal ischemia-reperfusion (IR) model. Under non-IR conditions, PLX treatment successfully depleted microglia in the retina. PLX suppressed the microglial activation response following IR as well as infiltration of monocyte-derived macrophages. This occurred in association with reduction of retinal expression of chemokines including *CCL2* and the inflammatory adhesion molecule *ICAM-1*. In addition, there was a marked suppression of retinal neuroinflammation with reduction in expression of *IL-1b*,* IL-6*,* Ptgs2*,* TNF-a*, and *Angpt2*, a protein that regulates BRB permeability. PLX treatment significantly suppressed inner BRB breakdown following IR, without an appreciable effect on neuronal dysfunction. A translatomic analysis of Müller glial-specific gene expression in vivo using the Ribotag approach demonstrated a strong suppression of Müller cell expression of multiple pro-inflammatory genes following PLX treatment. Co-culture studies of Müller cells and microglia demonstrated that activated microglia directly upregulates Müller cell-expression of these inflammatory genes, indicating Müller cells as a downstream effector of myeloid cells in retinal IR. Co-culture studies of these two cell types with endothelial cells demonstrated the ability of both activated microglia and Müller cells to compromise EC barrier function. Interestingly, quiescent Müller cells enhanced EC barrier function in this co-culture system. Together this demonstrates a pivotal role for myeloid cells in inner BRB breakdown in the setting of ischemia-associated disease and indicates that myeloid cells play a major role in iBRB dysregulation, through direct and indirect effects, while Müller glia participate in amplifying the neuroinflammatory effect of myeloid cells.

**Supplementary Information:**

The online version contains supplementary material available at 10.1186/s12974-024-03190-9.

## Background

Ischemic retinopathies including diabetic retinopathy (DR) and retinal vein occlusions (RVO) are major causes of vision loss globally [1, 2]. The most common cause of visual impairment in these conditions is macular edema, a result of increased retinal vascular permeability that leads to intraretinal and subretinal fluid [1–3]. Although anti-VEGF agents have led to improvements in visual outcomes, many patients do not fully respond to treatment. In addition, many patients require continued treatment over many years for both conditions, resulting in a large treatment burden [1, 2]. There is a great need for improved understanding of the causes and mechanisms regulating retinal vascular permeability, in order to enable development of additional treatments.

The blood-retinal barrier (BRB) plays an integral role in homeostatic maintenance of the retinal microenvironment through its selective regulation of flux of molecules from the systemic circulation [4, 5]. The BRB bears great similarity to the blood-brain barrier in its characteristics and critical function in regulating entry of blood-borne substances, including potentially harmful molecules, into the neural parenchyma [4]. The blood-retinal barrier has two distinct barrier systems, the inner and outer blood-retinal barriers [6, 7]. The inner blood-retinal barrier is primarily maintained by tight junctions of endothelial cells lining the retinal blood vessels, analogous to cerebral ECs of the blood-brain barrier [4, 5] and are critical to the health and integrity of the inner retina. The outer blood-retinal barrier (oBRB) is maintained by the retinal pigment epithelium (RPE), which regulates the transport of molecules between the choriocapillaris and the deep (outer) retina [6]. Integrity of the outer BRB is known to be critical, especially for diseases of the outer retina including age-related macular degeneration.

Breakdown of the inner BRB is a pivotal event in macular edema, the most common cause of visual impairment in ischemic retinopathies including DR and RVO [1, 3, 5]. Although compromise of retinal vascular endothelial cells, including its tight junctions, is recognized to be a critical proximal cause of vascular hyperpermeability, it is increasingly recognized that the pathophysiology of BRB dysfunction involves a broader breakdown of the neurovascular unit (NVU) [4, 8]. The cellular constituents of the retinal NVU include neurons and glial elements (Müller glia and astrocytes) in addition to vascular cells. Consequently, extensive research efforts are being dedicated to understanding the interactions between the cellular constituents of the NVU, in order to gain insights into mechanisms governing BRB health and disease.

Mononuclear phagocytes (MPs), or myeloid cells, have drawn increased attention as regulators of ischemic retinopathies and the retinal neurovascular unit [7, 9, 10]. In models of ischemia-induced retinal angiogenesis, MPs play a role in promoting pathologic neovessel formation [7, 11, 12]. With respect to the retina, MPs primarily comprise resident microglia and monocyte-derived macrophages from the systemic circulation [13]. Microglia are the principal resident immune cells of the retina and brain [14]. Although these cells play an important role in development and homeostatic maintenance, their dysregulation in disease contexts can result in retinal tissue damage and pathology, in part by recruiting monocyte-derived macrophages from the systemic circulation [9, 13, 14].

In order to gain insights into their potential pathogenic role in both systemic and ocular diseases, microglial depletion strategies have been of great interest, to gain insights into their role. A particularly useful approach for depletion has been pharmacologic inhibition of CSF-1R, an important survival factor for microglia. The agent PLX5622 (PLX) has been especially valuable, given its high selectivity for CSF-1R, excellent penetration into the CNS, and high efficiency of microglia depletion [15]. In part due to its ability to penetrate the blood-brain barrier, PLX has been used to gain insights into the role of both neurodegenerative and neuroinflammatory CNS diseases including Alzheimer [16] and experimental autoimmune encephalitis, a mouse model of multiple sclerosis [17, 18]. Similarly, this agent has been immensely useful for studying microglia in diverse retinal conditions including retinal degenerations and uveitis [19, 20]. More recent studies indicate that PLX also impacts function of systemic macrophages, especially with longer term treatment, so it is conceivable that the phenotypic effects of PLX treatment may be due at least in part on macrophages as well as microglia [21].

CSF-1R inhibition with PLX has been used to implicate the role of microglia/macrophages in the outer blood-retinal barrier, in a mouse model of endotoxin-induced uveitis (EIU) [22]. In this model, in which mice were subjected to repeated, systemic challenge with lipopolysaccharide (LPS), the authors demonstrated prominent subretinal fluid indicative of breakdown of the outer blood-retinal barrier. This was accompanied by prominent localization of Iba1 + cells in the subretinal space (in proximity to the subretinal fluid) and in the distal outer nuclear layer. CSF-1R inhibition suppressed the oBRB breakdown and subretinal fluid compared to vehicle. Notably, CSF-1R inhibition has not yet been reported in regard to inner BRB breakdown and ischemic retinal diseases.

Oxidative stress and inflammation are known to be critical drivers of disease progression of ischemic retinopathies [23]. Neuroinflammation is an important element of ischemic retinopathies. The mouse model of retinal ischemia-reperfusion is a model of ischemic injury involving the inner retina [24]. It involves pathophysiologic processes including oxidative stress and neuroinflammation in common with ischemic retinopathies including diabetic retinopathy. This model has been useful to study mechanisms of neurodegeneration [24] and inner blood-retinal barrier breakdown [25, 26]. In this study, we further characterized immune cell activation of mononuclear phagocytes in retinal IR and studied the impact of PLX treatment. Our depletion studies support the pivotal role of microglia/macrophages in inner blood-retinal barrier breakdown associated with retinal ischemia, in part by reprogramming of the Müller glia population.

## Materials and methods

### Animals

Animal studies were approved by the Institutional Animal Care and Use Committee of the Johns Hopkins University School of Medicine. All procedures involving animals were conducted under the Association for Research in Vision and Ophthalmology Statement for the Use of Animals in Ophthalmic and Vision Research. C57BL/6J mice, *Glast-Cre-ERT*(#012586) and RiboTag (B6J.129(Cg)-Rpl22tm1.1Psam/SjJ; catalog 029977) mice were purchased from the Jackson Laboratory. The animals were housed at 20–23 °C under 12:12 h light/dark cycles.

### Myeloid cell depletion with PLX5622

The CSF1R inhibitor PLX5622, which was provided by Plexxikon Inc. (USA), was formulated in AIN-75 A standard chow by Research Diets (USA). AIN-74 A standard chow served as a vehicle. For all retinal ischemia-reperfusion experiments, 8 week-old mice were fed with PLX5622 or vehicle diet starting at 7 days before retinal ischemia-reperfusion injury until each respective endpoint.

### Tamoxifen treatment

Tamoxifen was administrated as previously described [27]. In brief, tamoxifen (sigma T5648) was dissolved in corn oil (Sigma, USA, C8267) at a concentration of 7 mg/mL. Mice at P21 received a 3-day injection of 1 mg of tamoxifen and another 2-day injection of 2 mg of tamoxifen.

### Mouse model of ischemia-reperfusion (IR)

Mouse IR was performed as previously described [28]. In brief, mice were anesthetized with a cocktail of ketamine, xylazine, and acepromazine in PBS for 5–10 min to achieve deep anesthesia. A 30-gauge cannula needle attached to a line infusing sterile saline was inserted into the anterior chamber of the mice. Retinal ischemia was induced by increasing the intraocular pressure to 110 mmHg by raising the height of the sterile saline bottle. Ischemia was confirmed when the retina turned pale. After 90 min, the needle was removed. Mice were placed on a heating pad until they recovered from the anesthesia.

### Flow cytometry analysis

Flow cytometry was done as previously described [29]. Single-cell suspensions from the retina was obtained with the Neural Tissue Dissociation Kit (Miltenyi Biotec, US;130-093-231) and stained with APC anti-CD11b (eBioscience), FITC anti-CD45 (eBioscience), PE-Cy7 anti-CD86 (BD Biosciences), BV421 anti-CD206 (BioLegend), BUV 496 anti-CD192 (BD Biosciences), PE anti-Ly6G (BD Biosciences) and BUV 395 anti-CD11c (BD Biosciences) for 30 min. A 4-laser BD LSR II (BD Biosciences) was used to collect the data, and FlowJo software (BD Biosciences) was used for analysis. The gating strategy is presented in Fig. S1.

### Ribosome immunoprecipitation

Isolation of polysome-bound mRNA using RiboTag from tissues was performed as previously described ( [29]). After homogenization in 400 µL of polysome buffer (50 mM Tris, pH 7.5, 100 mM KCl, 12 mM MgCl2, 1% NP-40, 1 mM DTT, 200 U/mL RNasin, 1 mg/mL heparin, 100 µg/mL cycloheximide, and 1× protease inhibitor) using pellet pestles (Kimble Chase), retinal homogenates were centrifuged at 15,300 g for 10 min at 4 °C. 10 µl of the supernatant was collected for input. The rest of the supernatant was incubated with anti-HA antibody-conjugated magnetic beads (M180-11; MBL International) at 4 °C overnight on an orbital shaker. The next day, the beads were washed 4 times with high-salt buffer (50 mM Tris, pH7.5, 300 mM KCl, 12 mM MgCl2, 1% NP-40, 1 mM DTT, and 100 µg/mL cycloheximide). Beads were then resuspended in 350 µL RLT buffer plus β-mercaptoethanol, thoroughly vortexed, and pelleted using a magnetic separator. The supernatant was transferred into a new tube for RNA extraction with RNeasy Micro kit (QIAGEN). cDNA was synthesized with LunaScript^®^ RT SuperMix Kit (NEB #M3010).

### Real-time PCR analysis for retina tissue

RNeasy mini kit (QIAGEN) was used for RNA isolation from total retinas and cultured cells. Single-stranded cDNA was synthesized using M-MLV Reverse Transcriptase (Invitrogen) as previously described (59). Quantitative PCR was performed with SYBR Green PCR Master Mix (Invitrogen) with the StepOnePlus real-time PCR system (Applied Biosystems). *Gapdh* was used for normalization.

### Immunofluorescent staining

After fixation with 4% PFA and dehydration with 30% sucrose, eyes were embedded in an OCT compound solution. The cryo-blocks were sectioned using a cryo-microtome (10 μm sections) along the optic nerve. The sections were blocked with donkey serum and then stained with mouse anti-rabbit Iba1 (Wako), goat anti-mouse albumin (Novus), and rat anti-mouse CD31 (BD Biosciences) at 4 °C overnight. After washing 3 times, the sections were then incubated with secondary antibodies and DAPI. Photographs were taken using a Zeiss LSM 710.

### Leukostasis assay

Mice were deeply anesthetized and perfused with 37 °C of PBS for 3 min for removal of all nonadherent cells. Mice were then perfused over a span of 2–3 min with 5 ml rhodamine-labeled Concanavalin A (ConA; 40 µg/ml in PBS; Vector Labs) followed by 10 mL of PBS for 4 min. Eyeballs were then fixed in 4% PFA. The retinas were flat-mounted and visualized under confocal Zeiss LSM 710.

### Blood-retinal barrier (BRB) permeability assay

The blood-retinal barrier (BRB) assay was performed as described [30]. Mice were given an intraperitoneal injection of 1 µCi/1 g of [^3^H]mannitol (PerkinElmer, US). Retinas and part of the lung were collected and weighed 1 h after the injection. 1 mL NCSII solubilizing solution was added to each retina and lung tissue and incubated at 50 °C overnight. The next day, solubilized tissue was brought to room temperature and decolorized with 20% benzoyl peroxide in toluene at 50 °C. After returning to room temperature, 5 mL of scintillation fluid and 30 µL Glacial Acetic Acid were added to each vial. The samples were then stored at 4 °C overnight in the dark to eliminate chemoluminescence. Radioactivity was counted with a scintillation counter (LS 6500 Liquid Scintillation Counter; Beckman-Coulter, Indianapolis, IN, USA). The cpm of retina tissue and lung tissue were measured and normalized to the dry weight of each sample.

### Flash scotopic electroretinography (ERG)

ERG was performed as previously reported [31]. After dark-adaptation overnight, mice were anesthetized with ketamine (100 mg/kg) and xylazine (10 mg/kg), and pupils were dilated with topical tropicamide (1%). Scotopic ERG responses were measured using the Celeris ERG stimulator (Diagnosys, Lowell, MA) at flash intensities of 0.025, 0.250, 2.500, 7.900, and 79.050 cd*s/m^2^. ERGs were measured simultaneously from both eyes with a ground electrode placed into the forehead between the eyes and a reference electrode into the hip. 10 traces were averaged for the intensity of 0.025,0.250, 2.500, and 7.900 cd*s/m^2^, and 6 traces were averaged for the intensity of 79.050 cd*s/m^2^.

### Cell culture

Primary mouse Müller cells were isolated and cultured as previously described [32]. Eyeballs from C57BL/6J mice at P5 to P7 were removed and placed in serum-free DMEM (Thermo Fisher Scientific) plus 1× GlutaMAX supplement (Thermo Fisher Scientific) overnight at room temperature in the dark. The eyeballs were then incubated in a buffer containing trypsin, EDTA, and collagenase I at 37 °C. Retinas were dissected and placed in DMEM (high glucose) supplemented with 10% FBS, and 1× GlutaMAX. Retinas were minced into small pieces using forceps and incubated in the cell culture incubator for 3–4 days. Cultures were washed vigorously with the medium until only flat and firm adherent cell populations remained. Primary Müller cells were characterized by immunostaining with the Müller cell-specific markers glutamine synthetase (GS) and vimentin. Müller cells were used from passages 3 to 5.

BV2 cells were maintained in DMEM/F12 culture medium with 10% FBS. For the BV2 and Müller cell co-culture experiment, BV2 cells were seeded at a density of 0.5 × 10^5 on the Transwell inserts with a pore size of 0.4 μm (Corning #3470). After starvation with DMEM/F12 without FBS, the BV2 cells were treated with or without LPS (1 µg/ml) for 6 h. After rinsing with DMEM/F12 for 3 times, the inserts were then placed on the wells seeded with Müller cells and cultured for 48 h. After 48 h, the inserts were removed. The Müller cells were collected for RNA extraction.

### Endothelial cell solute flux assay

Endothelial cell solute flux assay was performed as previously described [33]. Human umbilical vein endothelial cells (HUVECs) were maintained in EGM2-MV culture medium (Lonza). For the solute flux assay, HUVECs were seeded at a density of 3 × 10^4^ on Transwell inserts (pore size 0.4 μm Corning #3470) which were pre-coated with fibronectin (Sigma). Once they were confluent, HUVECs were treated with EBM2 with 0.5% FBS overnight for starvation.

For the BV2 and Müller cell co-culture experiment, BV2 cells were seeded at a density of 0.5 × 10^5^ on Transwell inserts with a pore size of 0.4 μm (Corning #3470). BV2 cells were treated with or without LPS (1 µg/ml) for 24 h. After rinsing three times with DMEM/F12, the inserts were then placed over wells seeded with Müller cells in DMEM with 5% FBS and cultured for 72 h. After 72 h, inserts with BV2 were replaced with inserts with HUVECs and co-cultured for another 24 h. One insert without HUVECs served as blank control. 50 µl of 100 µM RITC-Dextran (Mol wt ~ 70,000; #9379, Sigma) were applied to the top inserts. 50 µl samples from the bottom chamber were collected at 30, 60, 90, 120,150, and 180 min. Additional 50 µl samples were collected from the inserts at 180 min. The fluorescein content of each sample was measured with FLUOstar OPTIMA (BMG Labtech GmbH). The diffusive permeability was calculated as previously reported [33].

### Statistical analysis

All bar graphs represent mean ± standard error (SEM). Unpaired, 2-tailed Student’s t-test was used for statistical analysis between 2 groups. One-way ANOVA was used to perform statistical analysis between 3 groups. Two-way ANOVA was used to perform statistical analysis between multiple groups of different treatments. Analysis was performed using GraphPad Prism, version 10. *p* < 0.05 was considered statistically significant.

## Results

### Myeloid cell activation and infiltration in the retinal ischemia-reperfusion (IR) model

**Fig. 1:**
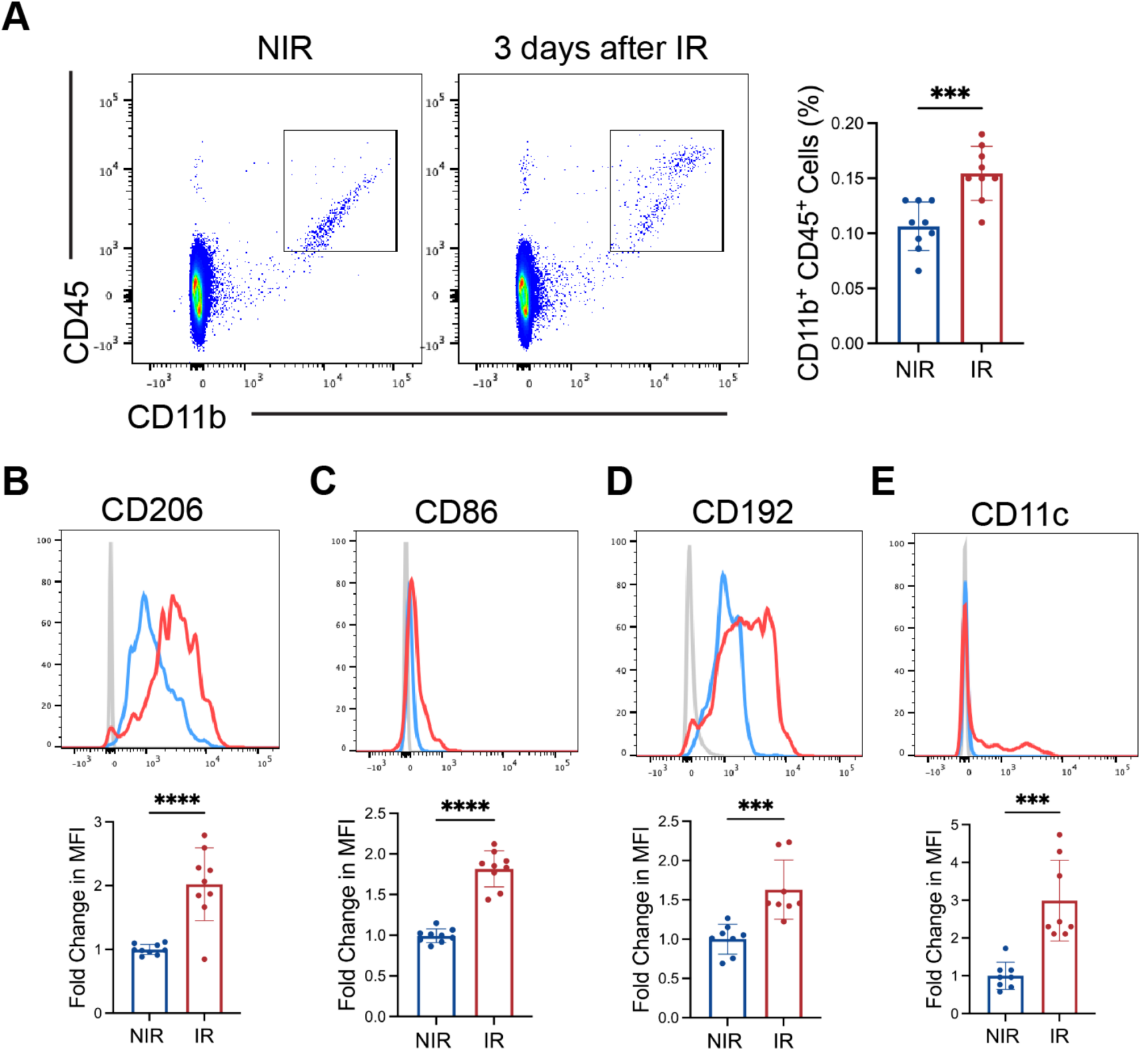
Myeloid cell infiltration and activation in the retinal ischemia-reperfusion model. **A** Flow cytometric analysis demonstrated increased Ly6G^−^ CD45^+^ CD11b^+^ myeloid cell numbers in the retina after 3 days of IR. **B** Increased expression of CD206, CD86, CD192, and CD11c on the Ly6G^−^ CD45^+^ CD11b^+^ myeloid cells at 3 days after IR. Red curve: IR; blue curve: NIR; grey curve: unstained blank control.  Data are shown as mean ± SEM, *n* = 6–9 mice per group, where each dot represents an individual eye. *p < 0.05 using Student’s *t* test

The rodent model of retinal ischemia-reperfusion has been a valuable model for gaining insights into ischemic retinopathies including diabetic retinopathy and vein occlusions [34, 35]. The pathophysiology of this model involves both oxidative stress and inflammation, exhibiting highly relevant pathology including blood-retinal barrier breakdown [25, 36] and neurodegeneration [37]. Previous studies have demonstrated the activation of immune cells in the IR model [38]. In this study, we further investigated immune cell activation in retinal IR. Using flow cytometric analysis, we observed an increased number of Ly6G^-^CD45^+^CD11b^+^ myeloid cells in the retina 3 days after IR injury, which constitutes both microglia and macrophages (Fig. 1A). Although CD45 expression can differentiate these two cell populations in non-disease settings, a recent study found that expression of CD45 in resident microglia cells was upregulated upon activation [39], limiting its use in differentiation of MPs in disease.

We further examined the expression of markers of myeloid cell activation and peripheral immune cell infiltration (Fig. 1B-E). We found an increased expression of CD206 and CD86 on the Ly6G^-^CD11b^+^ CD45^+^ cells. CD206 and CD86 were previously used as markers to indicate microglia/macrophage activation and polarization. The increased expression of CD206 and CD86 may indicate an increased activity of endocytosis/phagocytosis of the myeloid cells [40, 41]. We also observed increased expression of CD192, also known as CCR2, on the myeloid cells. CCR2 is expressed predominantly in the monocytes [42]. This finding suggests that the increased number of CD11b^+^ and CD45^+^ cells we observed includes infiltrating cells of monocytic origin. It should be noted that CCR2^+^ monocytes downregulate CCR2 expression after they migrate to the retina and differentiate into macrophages, making it difficult to distinguish the relative contributions of microglia and monocyte-derived macrophages to the mononuclear phagocyte population. Finally, we observed increased expression of CD11c. A previous study found that a subset of microglia responsible for phagocytic and proinflammatory responses expressed CD11c [43]. Overall, these findings suggest a significant activation of resident microglia as well as infiltration of peripheral myeloid cells into the retina after IR injury.

### CSR-1R inhibitor PLX5622 treatment reduced myeloid cell number and chemokine expression

**Fig. 2:**
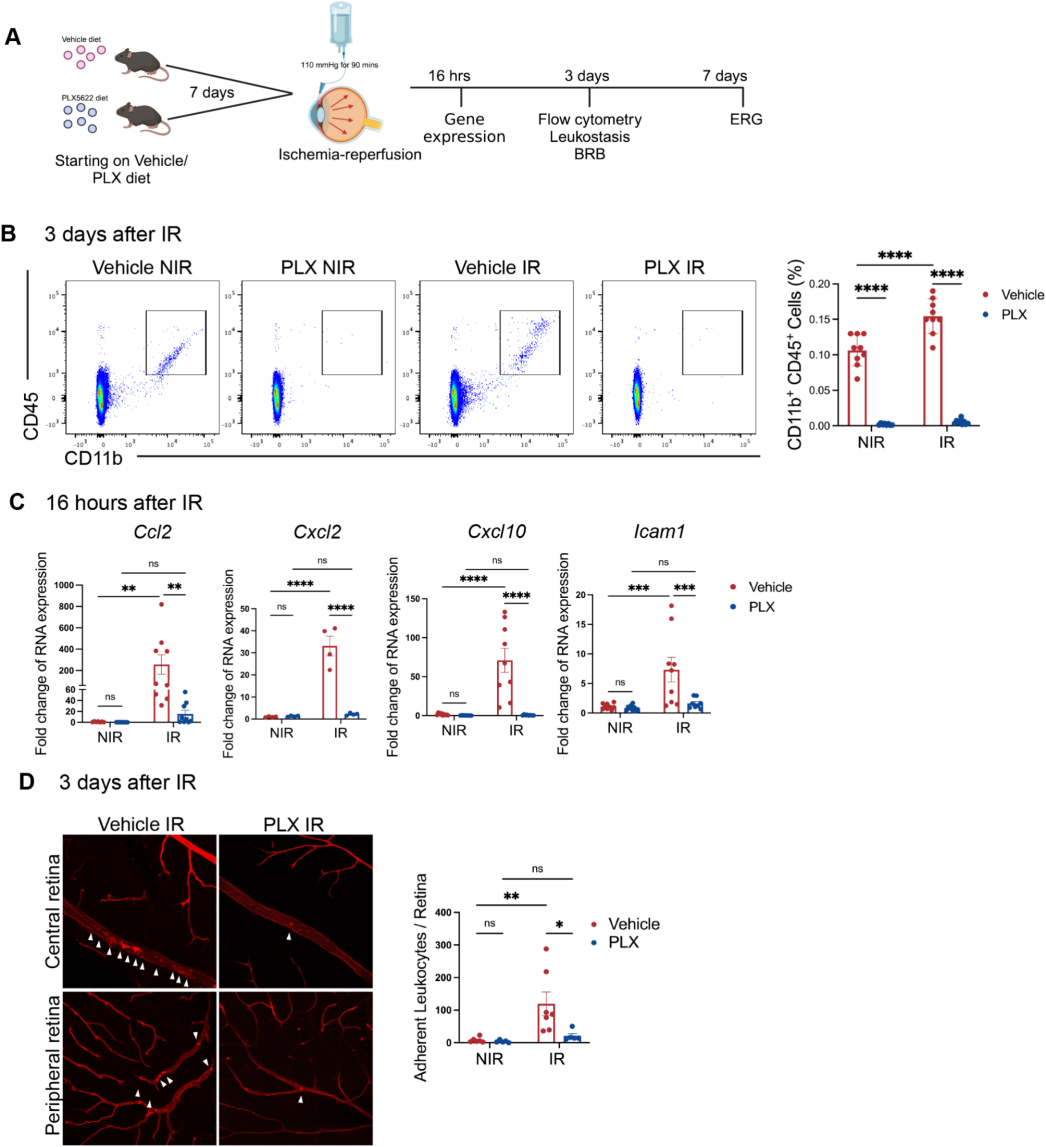
Depletion of myeloid cells in IR ameliorates inflammatory cell infiltration into the retina. **A** Schematic outlining the PLX5622 (PLX) treatment and experimental design in this study. Mice were fed with PLX or vehicle control chow for 7 days before the IR injury. **B** Flow cytometric analysis demonstrated a significant reduction of Ly6G^−^ CD45^+^ CD11b^+^ myeloid cells with PLX treatment at 3 days after the IR in both NIR eyes and IR eyes. **C** qPCR demonstrated reductions of chemokine and adhesion molecule expression in the PLX-treated retinas at 16 h after IR. **D** Leukocytes were labeled with Concanavalin A (ConA) in red. Inhibition of leukocyte adhesion (white arrowheads) in PLX-treated retina 3 days after IR. Data are shown as mean ± SEM, *n* = 4–9 mice per group, where each dot represents an individual eye. *p < 0.05, **p < 0.01, *** *p* < 0.001 using two-way ANOVA

In order to investigate the role of myeloid cells, we used a microglia depletion strategy with PLX5622 (PLX), a Colony-Stimulating Factor 1 Receptor (CSF1-R) inhibitor. As shown in Fig. 2A, mice were administered a diet with PLX or control vehicle (Vehicle) beginning 7 days before IR. After 3 days of IR, retinas were collected and analyzed with flow cytometry. The contralateral eyes with no IR injury served as NIR control. We first confirmed the efficiency of myeloid cell depletion with PLX in the NIR control eyes. As shown in Fig. 2B, there was a significant reduction of the Ly6G^-^CD11b^+^ CD45^+^ cells in retinas from PLX-treated mice. We then investigated the change in myeloid cell number in the IR eyes. As shown in Fig. 2B, we found that there was a significant reduction of the Ly6G^-^CD11b^+^CD45^+^ myeloid cells in the PLX-treated IR group compared to the CN-treated IR retinas. In addition, there was no difference in the myeloid cell number between the PLX-NIR and the PLX-IR groups. These findings suggest that inhibition with CSF-1R inhibitor PLX significantly depleted the myeloid cells, including both resident microglia and peripheral myeloid cells, in the retina after IR. In addition to the primary depletion of microglia, this effect of PLX treatment could be due either to a reduction in number of monocytes available to infiltrate into the retina or to the lack of microglia-mediated recruitment of systemic monocytes. Of note, we did not observe a change in monocyte number in spleens collected from the PLX-treated mice (Fig. S5), suggesting that the latter explanation may be more important cause.

Since there was a reduction of peripheral myeloid cell infiltration with the PLX treatment, we next looked into the expression change of chemokines and adhesion molecules in retinas with PLX treatment after IR. As expected, I/R induced a significant increase in the expression of chemokines including *Ccl2*, *Cxcl2*, and *Cxcl10* and the adhesion molecule *Icam1* after 16 h of IR. PLX treatment significantly reduced the expression of these genes in the retina after IR (Fig. 2C). This finding suggests that the depletion of myeloid cells led to a reduction of both chemokine and adhesion molecule expression in the retina, thereby ameliorating inflammatory cell infiltration into the retina.

We next performed a leukocyte adhesion assay at 3 days after IR. As shown in Fig. 2D, IR resulted in a dramatic increase in leukocyte adhesion, which was observed in blood vessels from both the central and peripheral retina. PLX treatment significantly suppressed IR-induced leukostasis.

### Myeloid cell depletion ameliorated retinal neuroinflammation induced by IR

**Fig. 3:**
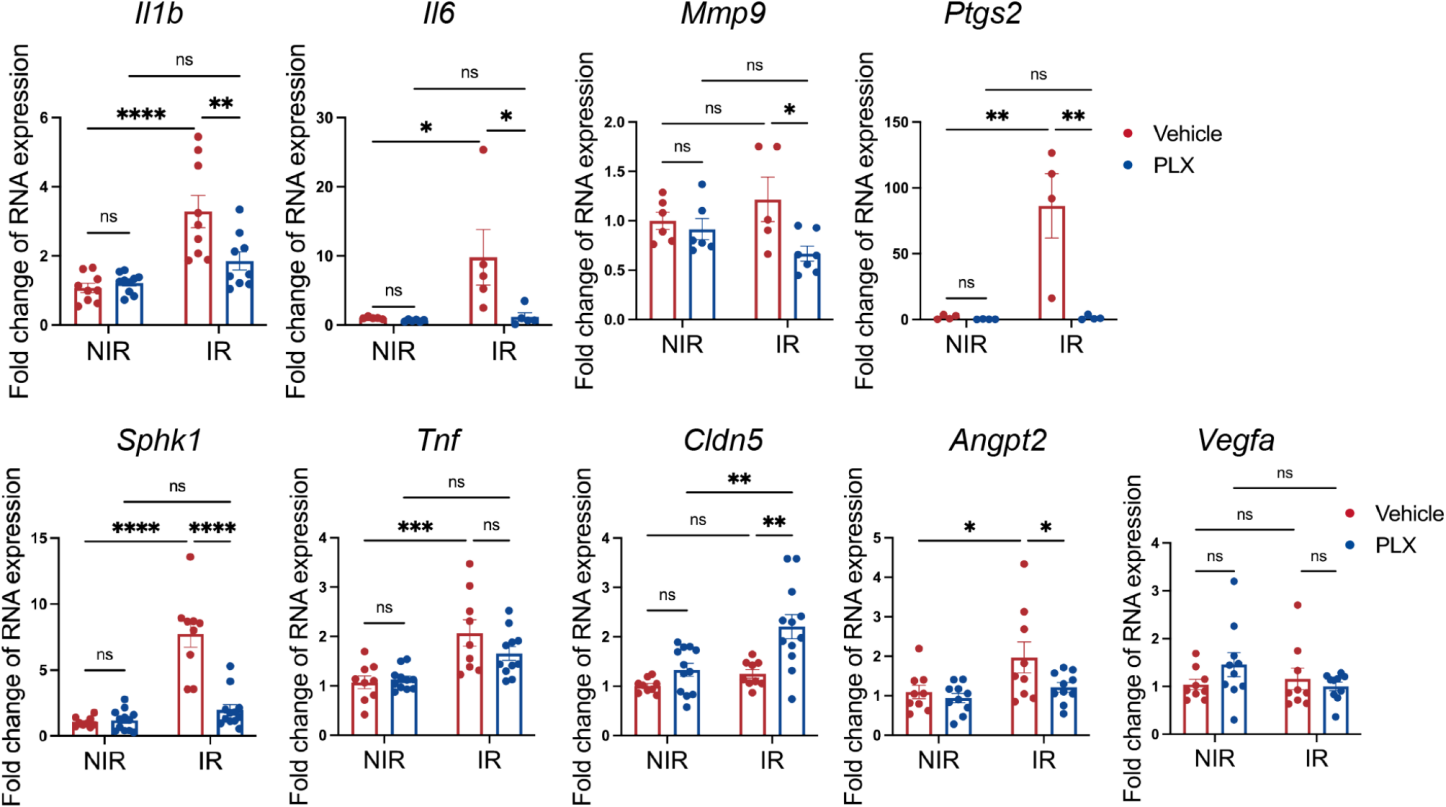
Myeloid cell depletion regulated inflammatory gene expression in IR. qPCR analysis from the total retina demonstrated that IR induced expression of multiple inflammatory genes, including *Il1b*,* Il6*,* Ptgs2*,* Sphk1*, and *Tnf*. PLX treatment significantly reduced the expression of *Il1b*,* Il6*,* Ptgs2*, and *Sphk1*, but not *Tnf*. PLX treatment also increased the expression of *Cldn5*, a major endothelial tight junction protein, and *Angpt2*, an important regulator of endothelial cell permeability. Neither IR nor PLX treatment affected *Vegfa* expression. Data are shown as mean ± SEM, *n* = 5–12 mice per group, where each dot represents an individual eye. *p < 0.05, **p < 0.01, **** *p* < 0.0001, ns: *p* > 0.05 using two-way ANOVA

We studied the effect of myeloid cell depletion with PLX on retinal gene expression in IR. We first investigated the expression of inflammatory cytokines in the total retina after IR. At 16 h after IR, there was a significant increase in the expression of multiple inflammatory genes, including *Il1b*,* Il6*,* Ptgs2*,* Sphk1*, and *Tnf*. PLX-treated retinas exhibited significant reduction in the expression of *Il1b*,* Il6*,* Ptgs2*,* Sphk1*, and *Mmp9* compared to the CN-treated retinas. Interestingly, we only observed a trend of reduction of *Tnf* in the PLX-treated retinas after IR. This finding might indicate that cell types other than myeloid cells, such as endothelial cells and neurons, could also express *Tnf* under the IR condition. PLX treatment did not affect *Vegfa* expression in the retina. Consistent with a previous report [36], we did not observe any changes in *Vegfa* mRNA expression induced by IR. However, we did observe increased expression of *Angtp2*, the gene coding for Angiopoietin 2, an important regulator of the BRB [44, 45]. Depletion of myeloid cells with PLX significantly reduced the expression of Angpt2. Claudin-5 protein is a key protein that regulates the endothelial cell tight junction. We found that *Cldn5* gene expression was not changed 16 h after IR. However, PLX treatment increased expression of *Cldn5* under the IR condition. This finding suggests the possibility that myeloid cell depletion could directly regulate the endothelial cell tight junction and the blood-retinal barrier function (Fig. 3).

### Depleting myeloid cells with PLX5622 ameliorated blood-retinal barrier breakdown in IR

**Fig. 4:**
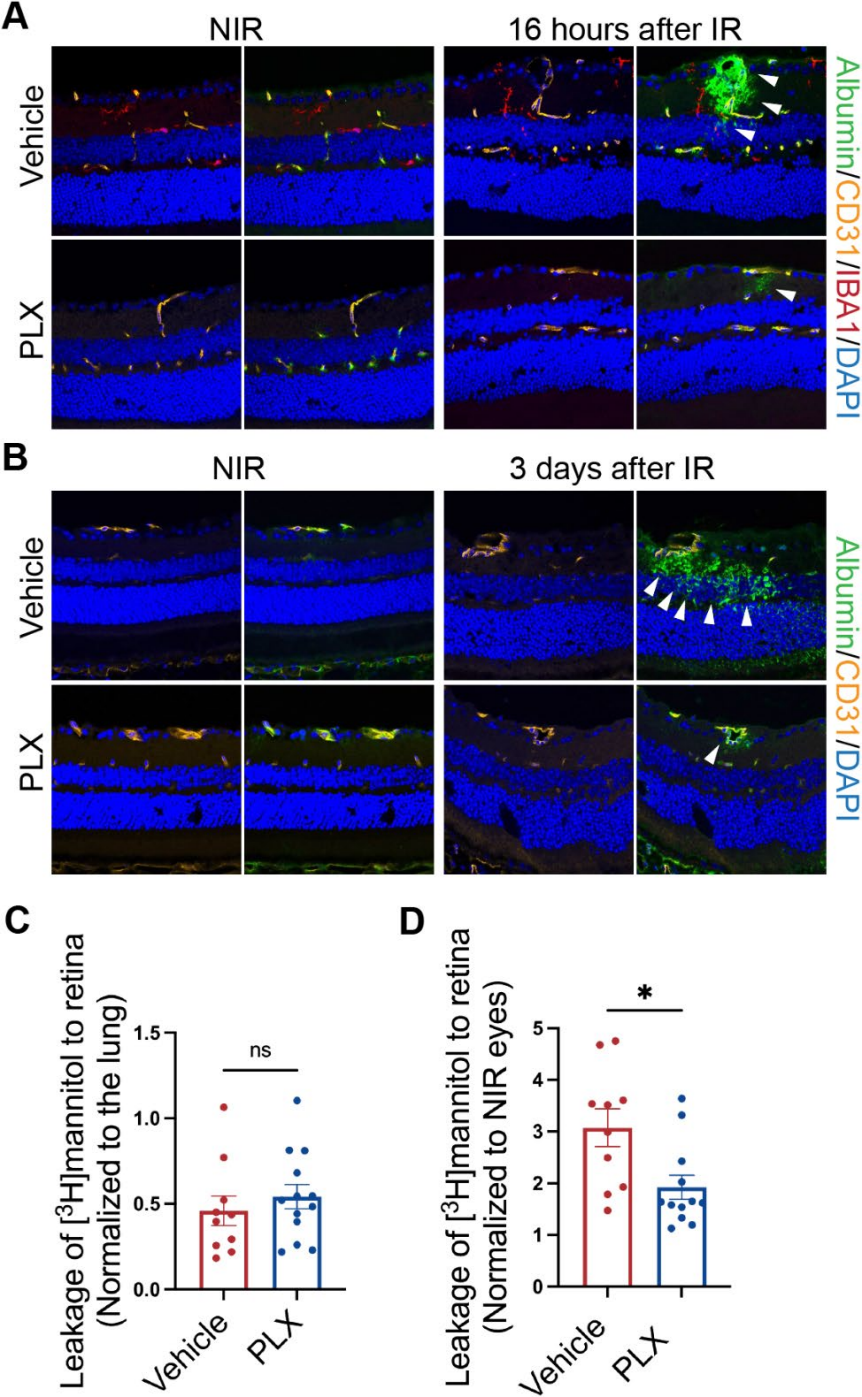
Myeloid cell depletion ameliorated inner blood-retinal barrier breakdown in IR. **A** and **B** Immunofluorescence staining of retinal cryosections demonstrated that there was reduced albumin (green) leakage outside the CD31^+^ vessel (yellow) in the PLX-treated retina at 16 h (**A**) and 3 days (**B**) after IR. White arrowheads indicate albumin extravasated from blood vessels; **C** PLX treatment did not affect inner blood-retinal barrier permeability in the NIR eyes examined by the leakage of H^3^-mannitol in the retina after systemic administration. **D** Depletion of myeloid cells with PLX significantly reduced inner blood-retinal barrier breakdown at 3 days after IR. Data are shown as mean ± SEM, *n* = 10–13 mice per group, where each dot represents an individual eye. *p < 0.05, ns: *p* > 0.05 using Student’s *t* test

Based on the expression change of inflammatory cytokines as well as *Angpt2* and *Cldn5* with PLX treatment, we further investigated whether PLX affected inner blood-retinal barrier (iBRB) permeability in IR. We first evaluated retinal vascular permeability change with immunofluorescence (IF) staining of albumin on retinal cryosections. Under physiological conditions, albumin is localized within blood vessels in the retina [46, 47]. As shown in Fig. 4A-B, albumin (green) colocalized with the CD31^+^ vessels (yellow) in both CN- and PLX-treated eyes under the NIR condition, which suggested that the depletion of myeloid cells under normal conditions did not affect retinal vascular permeability. At 16 h after IR, there was a notable increase in albumin extravasation outside the retinal vessels (white arrowheads) in the CN-treated group (Fig. 4A, right panel). There was a marked reduction in albumin leakage in PLX-treated retinas compared to the CN-treated group. Furthermore, even at 3 days post-IR, the PLX-treated retinas continued to exhibit a notably smaller area of leakage as depicted in Fig. 4B (right panel). IBA1 staining (red) also indicated the depletion of myeloid cells with PLX treatment (Fig. 4A, Fig. S2) .

To further confirm our findings from albumin immunofluorescence staining, we quantified the BRB breakdown by measuring the extent of leakage of H^3^ labeled-mannitol into the retina 3 days after IR. As shown in Fig. 4C, we did not observe any difference between the PLX- and CN-treated eyes under NIR conditions. Consistent with the immunofluorescence staining results for albumin, a significant reduction of H^3^-mannitol leakage was observed in retinas of PLX-treated eyes at 3 days after IR. These findings suggest that the depletion of myeloid cells ameliorated the inner BRB breakdown induced by IR.

### Myeloid cell depletion did not affect neuronal degeneration in IR

**Fig. 5:**
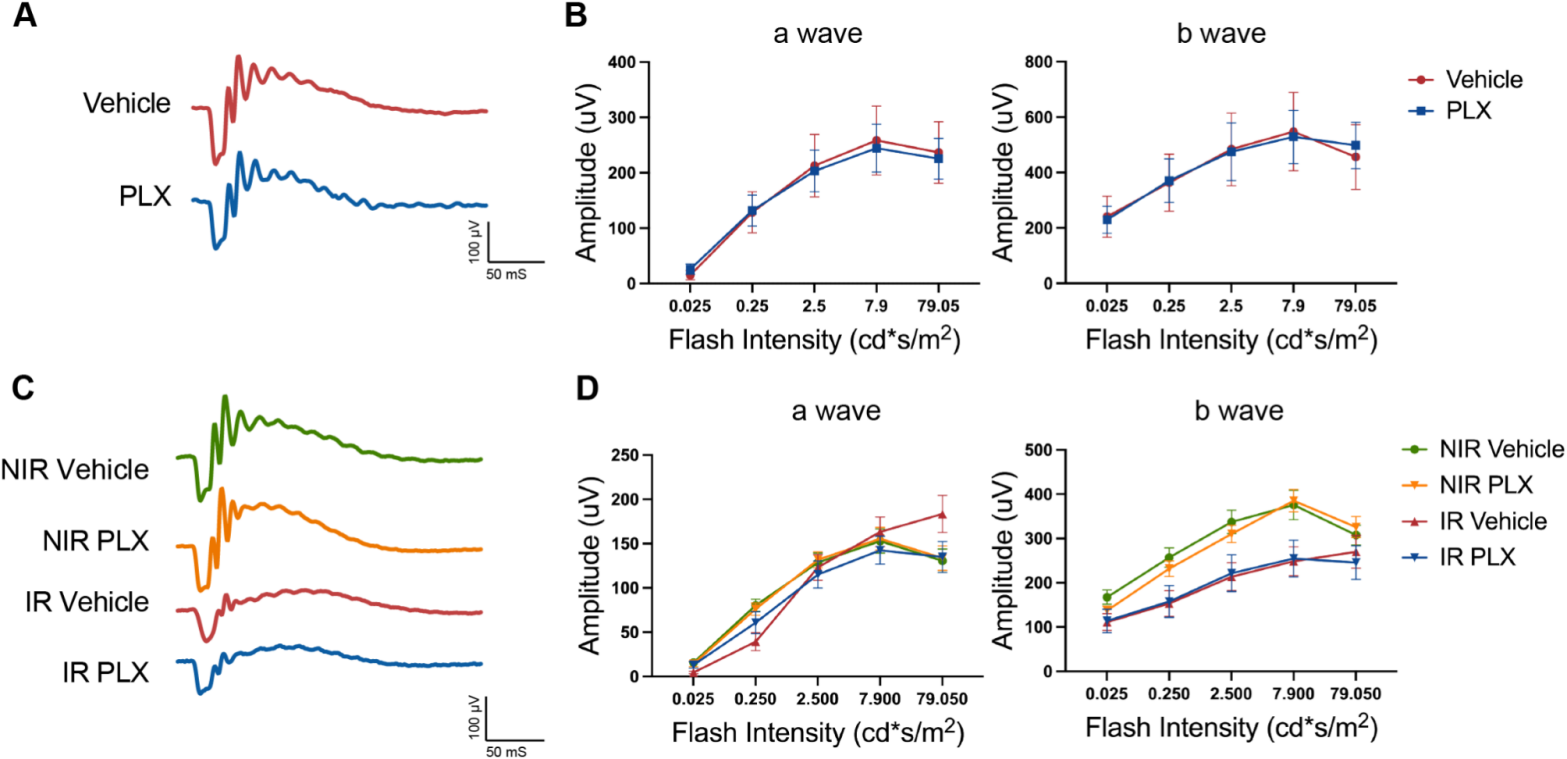
Myeloid cell depletion did not affect neuronal dysfunction 7 days after IR. **A** Representative ERG tracings with flash intensity of 7.9 cd*s/m^2^ from the vehicle- or PLX-treated mouse retina after 7 days of treatment. 7-day treatment with PLX did not affect baseline a-wave and b-wave compared to the vehicle-treated group. **B** Representative ERG tracings from IR and NIR mice with vehicle or PLX treatment at 7 days after IR. There was no difference between PLX-treated and vehicle eyes on ERG a-wave and b-wave. Data are shown as mean ± SEM, *n* = 10–13 mice per group

We further evaluated whether the depletion of myeloid cells affects neuronal function in IR using electroretinography (ERG). It has been reported that myeloid cell depletion with PLX might reduce the retinal function as assessed by ERG in some contexts [48]. We tested the baseline ERG before IR to exclude the possibility that PLX might affect the ERG function before IR injury. As shown in Figs. 5A and B and 7 days of PLX treatment did not affect the a-wave or b-wave of the ERG. We found that IR significantly reduced the ERG b-wave amplitude, but not the a-wave (Fig. 5D). Treatment with PLX did not affect either the ERG a-wave or b-wave under the IR condition. This finding indicates that depletion of myeloid cells did not overtly affect neuroretinal function or neuronal degeneration in IR. It has been reported that long-term depletion of myeloid cells with PLX can adversely affect retinal neuronal function assessed by ERG [49]. The lack of observed effect in our study could be due to the shorter duration of depletion.

### Absence of myeloid cells ameliorated inflammatory gene expression of Müller glia in IR

We next investigated the effect of myeloid cell depletion on Müller cells. Müller cells surround retinal blood vessels [8]. Under disease conditions, Müller cells secrete VEGF and other inflammatory cytokines, which can lead to the breakdown of the iBRB. The cross-talk between Müller cells and myeloid cells in neurodegeneration has gained increasing attention in recent years. The role of interaction between myeloid cells and Müller cells in regulating the inner BRB function is less clear.

**Fig. 6:**
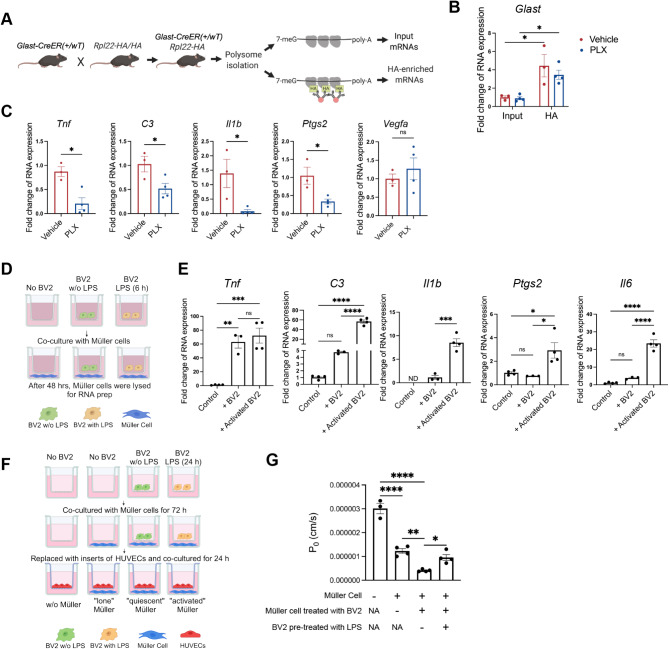
Myeloid cells regulate Müller glia activation in IR. **A** Schematic describing the experimental design of GlastCreER Müller specific-RiboTag mice. **B** Enrichment of Müller cell translatome was confirmed with the qPCR analysis of *Glast (*glial high affinity glutamate transporter) gene expression with Input and HA-enriched samples. **C** qPCR analysis showed that PLX treatment significantly reduced the expression of *Tnf*,* C3*,* Il1b*, and *Ptgs2* genes on Müller cells after IR. PLX treatment did not affect the Müller cell *Vegf* expression. **D** Experimental design of BV2 and Müller cell culture. **E** qPCR analysis demonstrated increased gene expression of *Tnf*,* C3*,* Il1b*,* Ptgs2*, and *Il6* in Müller cells when co-cultured with BV2 cells. **F** Experimental design of BV2, Müller cell, and HUVEC co-culture. **G** HUVECs co-cultured with Müller cells (“lone” Müller cells), or with BV2 previously cocultured with Müller cells (“quiescent” Müller cells), exhibited reduced diffusive solute flux. HUVECs co-cultured with Müller cells previously co-cultured with LPS-treated BV2 (“activated” Müller cells) exhibited increased diffusive solute flux. Data are shown as mean ± SEM. For Fig. 6B and C, *n* = 3–4 mice per group, where each dot represents an individual eye. *p < 0.05, ns: *p* > 0.05 using Student’s *t* test; For Fig. 6E and G, *n* = 3–4 per group, where each dot represents an individual sample. *p < 0.05, **p < 0.01, *** *p* < 0.001, **** *p* < 0.0001, ns: *p* > 0.05 using one-way ANOVA. ND: non-detectable

To explore whether depletion of myeloid cells impacts the iBRB under IR conditions through interactions with Müller cells, we generated *GlastCreER; Rpl22* mice (Fig. 6A). GlastCreER mice express CreER under the control of the *Glast* (glial high affinity glutamate transporter) promoter. When crossed with the Rpl22 mice, it enables analysis of the translatome in Müller cells. We first confirmed the enrichment of Müller cell-specific RNAs in the HA-pulldown samples by examining *Glast* expression (Fig. 6B). We then investigated the expression of inflammatory factors in Müller cells with PLX treatment. Expression of many inflammatory factors such as *Il1b* and *Ptgs2* was barely detectable under NIR conditions and strongly upregulated in IR in the Müller enriched samples (Fig. S3). Under IR conditions, we found that PLX treatment significantly reduced the expression of inflammatory genes, including *Tnf*,* Il1b*,* C3*,* and Ptgs2* (Fig. 6C). We did not observe any change in the *Vegf* expression. These data suggest that depleting myeloid cells ameliorated the expression of inflammatory genes in Müller cells induced by IR.

To further confirm the direct effect of myeloid cells on Müller cells, we performed in vitro experiments with microglia and Müller cell co-culture (Fig. 6D). After co-culturing for 48 h, Müller cells were collected and analyzed using qPCR. We found that co-culturing of Müller cells with BV2 microglia leads to an increase in the expression of *Tnf*. When co-cultured with LPS-activated BV2 cells, there was a significant upregulation of *C3*,* Il1b*,* Ptgs2*, and *Il6* in the Müller cells (Fig. 6E). These findings are consistent with the in vivo Müller cell RiboTag experiment and suggest that activated myeloid cells can regulate the expression of inflammatory factors in Müller cells, contributing to and further accentuating iBRB breakdown in the retina.

We further investigated whether myeloid cells affect endothelial cell barrier function directly or indirectly (through regulation of Müller cells) using an in vitro endothelial cell assay of diffusive solute flux [33]. We found that culture medium collected from LPS-treated BV2 significantly reduced barrier function of cultured human umbilical vein endothelial cells (HUVEC) compared to culture medium collected from BV2 without LPS (Fig. S3). This finding indicates that activated microglia can directly impair endothelial cell barrier function. We next investigated whether microglia can regulate EC barrier function indirectly via Müller cells. We first cultured Müller cells under three conditions – in the absence of BV2 microglia (“lone” Müller cells), in co-culture with untreated BV2 microglia (“quiescent” Müller cells), and in co-culture with LPS-treated BV2 (“activated” Müller cells). We then removed the microglia and co-cultured the differentially treated Müller cells with HUVECS (Fig. 6F-G). Interestingly, EC barrier function was enhanced when co-cultured with “lone” Müller cells. In addition, EC barrier function was further strengthened in co-culture with “quiescent” Müller cells that had been exposed to quiescent microglia as compared to “lone” Müller cells. This suggests that quiescent microglia might influence Müller cells toward a phenotype that enhances EC barrier function. Strikingly, this beneficial effect on EC barrier function was lost when ECs were co-cultured with “activated” Müller cells that had been exposed to LPS-activated microglia. Together, these findings indicate that microglia can regulate vascular cell barrier function both directly and indirectly through the regulation of Müller cells.

## Discussion

Breakdown of the inner blood-retinal barrier is known to be the critical instigator for macular edema, the most common cause of visual loss in ischemic retinopathies. Intensive research interest has emerged in gaining insights into the regulation of retinal vascular endothelial integrity by other cellular elements of the retina. In this current study, we found that the depletion of myeloid cells with the CSF1R inhibitor PLX5622 (PLX) ameliorated the inner blood-retinal barrier breakdown induced by ischemia-reperfusion. This finding indicates that microglia/macrophages are important in regulating the inner BRB under disease conditions, highlighting myeloid cell modulation as a promising therapeutic strategy.

Pharmacologic targeting of CSF1R has been a valuable investigational approach for studying the role of microglia, including in the brain and eye. The CSF1R inhibitor PLX has been especially useful, based on its high selectivity and ability to penetrate the blood-retinal and blood-brain barriers [15]. It accomplishes highly efficient depletion of retinal microglia, achieving 98% depletion after 1 week of treatment [50]. In the current study, we found that 7-day treatment with PLX markedly reduced the number of CD11b^+^ and CD45^+^ cells in the retina in both non-IR and IR eyes. This indicates a striking reduction of both retinal microglia and infiltrating monocyte-derived macrophages from the blood following ischemia-reperfusion injury. Notably, our qPCR analysis at 16 h after IR injury demonstrated that PLX treatment resulted in marked downregulation of multiple genes involved in recruitment of inflammatory cells from the circulation, including the adhesion molecule ICAM-1 and chemokines such as CCL2. CCL2 is known to be an important chemokine for macrophage recruitment into the retina during inflammation [51, 52]. Since microglia are known to secrete and/or regulate these molecules [53], our data are consistent with the concept that PLX treatment suppresses the reactive microglial response to IR and with it, a microglial-medicated impetus for recruitment of other inflammatory cells from the circulation, including monocyte-derived macrophages. This would be similar to the implication of microglia as a critical initiator of peripheral immune cell infiltration in an animal model of experimental autoimmune uveitis [19]. It is important to be mindful that PLX treatment, in addition to its effect on depleting resident microglia in the CNS and retina, can also affect the immune cell population such as macrophages and dendritic cells in peripheral blood and other tissues/organs such as the spleen, bone marrow, and skin [21]. This effect is especially apparent for longer durations of PLX treatment compared to shorter term treatment [18, 21]. With our shorter-term regimen of PLX treatment, we did not observe a significant effect on monocyte numbers in the spleen. Nevertheless, we cannot complete exclude the possibility that a reduction in systemic, circulating monocytes could be a contributing factor to the reduction in infiltrating mononuclear phagocytes following IR with PLX treatment.

The retinal ischemia-reperfusion model directly impairs the retinal vascular circulation and has therefore yielded many important insights relating to this vascular bed. Inner BRB breakdown has been well-characterized in this model [36]. With its primary effect in suppressing microglia as well as modulating macrophages, we used PLX to study and implicate the role of these cell populations. We observed that depleting myeloid cells with the CSF1R inhibitor PLX ameliorated the inner BRB breakdown, using two independent methods for evaluating retinal vascular leakage. We found that the lack of myeloid cells ameliorated the BRB breakdown, suggesting that immune cells are an important factor inducing the iBRB breakdown. In contrast, we did not observe any neuronal protection effect from myeloid cell depletion in this IR setting, suggesting a specific effect on vascular regulation. Our study could indicate a primary effect of microglia, or alternatively a combined effect of microglia and macrophages. This is consistent with previous studies of microglia/macrophage modulation. Agents that are known to modulate microglia and macrophages, including minocycline and the GLP-1R agonist exendin-4, inhibited inner blood-retinal barrier breakdown after retinal ischemia-reperfusion [25, 54]. Interestingly, minocycline treatment suppressed neuroinflammation and had a selective effect in protecting against retinal vascular permeability, without impacting neurodegeneration [25], similar to our own findings. In the STZ-induced diabetic rodent model, depletion of microglia (and possibly macrophages) alleviated neuronal and vascular damage in the STZ-induced diabetes model [55]. AAV-mediated delivery of the anti-inflammatory chemokine fractalkine in the STZ-diabetes model, reduced fibrinogen leakage from retinal vessels. This was associated with regulation of microglia-mediated neuroinflammation [56, 57].

Our study indicates the importance of myeloid cells, also referred to as mononuclear phagocytes, in the breakdown of the inner blood-retinal barrier, in the context of ischemia-associated injury. Notably, PLX-mediated depletion was previously investigated in a study of outer blood-retinal barrier breakdown in a mouse model of endotoxin (LPS)-induced uveitis (EIU) [22]. In this study, mice were subjected to repeated, systemic challenge with lipopolysaccharide (LPS). As noted by the authors, the primary BRB defect in these mice was at the level of the retinal pigment epithelium (RPE), the site of the outer blood-retinal barrier. This was manifested by the marked increase in subretinal fluid in these LPS-challenged mice. This was associated by fluorescein angiographic studies that showed focal, non-vascular leakage accounting for the subretinal fluid. The authors also observed an increase in retinal vascular permeability that they found to be transient in this model. The study reported that PLX treatment, with its modulation of mononuclear phagocytes, suppressed the outer blood-retinal barrier breakdown. LPS is known to be a strong, direct activator of microglia and macrophages [58, 59], which could be an important mechanism for the oBRB dysregulation. This provides important evidence supporting the ability of the mononuclear phagocytes to regulate RPE-mediated barrier function.

A critical concept in the understanding of retinal vascular disease is the pathogenic role of dysfunction of the neurovascular unit, comprising neurons and glial elements in addition to vascular cells [8]. Microglia play an important role in regulating these cellular elements both in the retina and brain. In neurodegenerative diseases of the brain, there is increasing awareness of the importance of crosstalk between microglia and astrocytes, including the role of microglia in initiating and modulating astrocytic responses to stressors [60–62]. Strikingly, although retinal microglia are increasingly appreciated to play significant regulatory roles in multiple retinal diseases [53], its crosstalk with glial elements promoting disease progression remains largely undefined, including in the setting of retinal ischemia-reperfusion [7, 34]. In the retina, Müller cells are the predominant glial element, representing 90% of the glia. Müller cell reactivity occurs in retinal disease settings, and these reactive Müller glia can exert both neuroprotective and neurotoxic effects [63, 64]. Müller cells are known to be an important cell type that contributes to the inflammatory environment in ischemic retinopathies, especially with respect to their production of cytokines. Müller cells envelope retinal blood vessels [8], facilitating their regulation of the vasculature. Müller cells have been implicated as an important regulator of neuroinflammation in diabetic retinopathy and vascular leakage, in large part through its regulation of the growth factors and cytokine environment. Interestingly, Müller cells can have both pathogenic [65, 66] and protective [67] effects in the retina, including vascular barrier function, depending on the context and the activation state the Müller cells. In order to study the effect of microglia depletion on Müller cell state, we used a Ribotag approach that allowed us to analyze cell-specific gene expression in vivo [29, 68]. For Müller cell-specific expression, we used Glast-CreER transgenic mice, which allows for Müller cell-specific expression [27]. Strikingly, we found that PLX treatment significantly curtailed Müller cell expression of *Tnf*,* Il1b*,* C3*, and *Ptgs2*, which encodes COX-2. In contrast, there was no effect on *Vegfa* expression. This indicates that reactive microglia and macrophages can provoke an inflammatory response in Müller cells in retinal IR. As an additional study, we used an in vitro co-culture system of microglia and Müller cells. This demonstrated that activated microglia similarly upregulated Müller cell expression of multiple pro-inflammatory genes compared to quiescent microglia. Notably, Müller cell expression of *Tnf was* significantly upregulated by both quiescent and activated microglia in this in vitro system. We also investigated the effect of culture microglia and Müller cells in regulating endothelial cell barrier function. Interestingly, we found that when co-cultured with unperturbed Müller cells, the barrier function of endothelial cells was strengthened. This barrier function was further enhanced when the endothelial cells were co-cultured with Müller cells which had been previously co-cultured with quiescent BV2 microglia. These findings suggest that Müller cells and microglia can be beneficial in supporting the barrier function of endothelial cells. However, this beneficial effect on EC barrier function was lost when ECs were co-cultured with “activated” Müller cells that had been exposed to LPS-activated microglia. This supports the ability of reactive microglia to directly activate Müller cells toward a pathogenic state, further impairing endothelial cell barrier function.

It should be noted that reactive microglia likely regulate other cell types in the context of retinal IR, for instance vascular cells. For instance, we observed that PLX treatment resulted in an upregulation of *Cldn5* in IR. This endothelial cell-specific gene encodes the tight junction protein CLDN5, an important constituent of the iBRB [4].This could be due to the suppression of inflammatory cytokines by myeloid cell depletion, including TNF-a and IL-1b which are known to induce expression of *Cldn5* [69, 70]. We also observed that the expression of angiopoietin 2 was significantly reduced with myeloid cell depletion under the IR condition. Ang2, expressed by multiple retinal cell types including vascular endothelial cells, mural cells, and neurons, plays an important role in directly regulating EC barrier function in the retina.

It is also worthwhile to consider the potential involvement of other cell types in the neurovascular unit. With respect to macroglia, we focused on Müller cells, since they are the predominant glial element of the retina and are well-known to regulate the inner blood-retinal barrier [71]). Astrocytes have received much less attention for their role in iBRB regulation, although they are known to play an essential role in developmental retinal angiogenesis of the superficial retinal vascular plexus [72]. The retinal vessels of the superficial retinal plexus are surrounded by the specialized feet of Müller cells and astrocytes, while vessels of the intermediate and deep plexi are only surrounded by Müller cells [71]. Therefore, it will be of interest to consider the potential involvement of astrocytes in future studies.

Finally, we should note the emerging research on perivascular macrophages as additional resident mononuclear phagocytic cell type in the retina. It has been suggested that these cells play a role in the maintenance of the blood-retinal barrier [73]. In addition, their role in retinal disease pathology is increasingly appreciated, including the promotion of inflammation in model of blue light-induced photoreceptor degeneration [74]. Therefore, it will be of great interest in future studies to investigate the role of perivascular macrophages as an additional important myeloid cell type involved in regulating the inner blood-retinal barrier. Overall, our study supports a central role for microglia and possibly monocyte-derived macrophages as mediators of inner blood-retinal barrier breakdown, indicating an important cellular target of therapeutic modulation of macular edema in a variety of ischemic retinal conditions.

## Conclusion

**Fig. 7:**
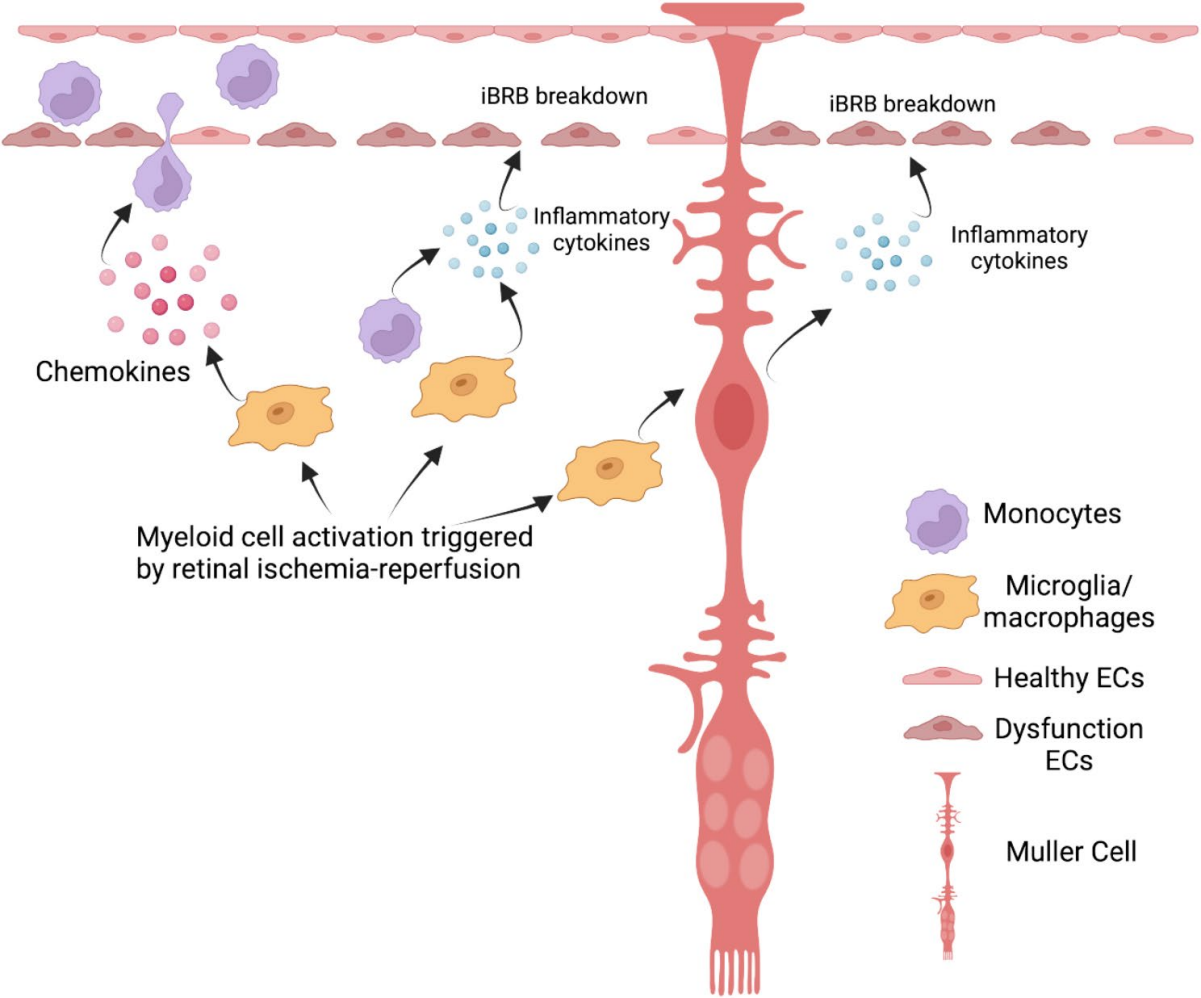
Schematic of the proposed role and effect of microglia and mononuclear phagocytes in retinal ischemia-reperfusion. Retinal microglia are activated by IR stress. Reactive microglia secrete chemokines, recruiting monocyte-derived macrophages infiltrating from the retinal circulation. Activated myeloid cells (microglial and monocyte-derived macrophages) express inflammatory cytokines that promote inner blood-retinal barrier breakdown. In addition, microglia (and possibly monocyte-derived macrophages) modulate retinal Müller glia, promoting a neuroinflammatory phenotype including secretion of inflammatory cytokines by this glial population, further exacerbating inner blood-retinal barrier breakdown. Müller cells thereby play an important pathogenic role in amplifying the initiating effect of microglial activation

The present study demonstrates the importance of mononuclear phagocytes as mediators of inner blood-retinal barrier breakdown in the mouse model of retinal ischemia-reperfusion. PLX5622 treatment efficiently depletes microglia in healthy retinas, with suppression of the microglia activation response to retinal IR as well as of infiltration of monocyte-derived macrophages. This results in constraint of the overall neuroinflammatory response to IR as well as IR-induced retinal vascular hyperpermeability. An important aspect of the effect is highly likely to be the direct regulation of retinal microglia. In addition, it likely also reflects the regulation of downstream effects of microglia, including the stimulation of infiltrating monocyte-derived macrophages from the circulation. An additional important effect is on microglia-induced activation of Müller glia toward a pathogenic state. These pathogenic Müller cells play an important role in amplifying the initiating effect of microglial activation. Our proposed conceptualization of the role of microglia in triggering the neuroinflammatory response and iBRB breakdown is depicted in Fig. 7.

### Electronic supplementary material

Below is the link to the electronic supplementary material.

**Supplementary Fig. 1**. Gating strategy for retinal flow cytometric analysis. Ly6G-positive cells were excluded prior to analysis of CD11b CD45 expression.

**Supplementary Fig. 2**. Immunofluorescence staining of IBA1 in retinas collected after 3 days of IR. Myeloid cells were stained with IBA1 in red. There was a significant increase in IBA1 + cells in the IR Vehicle group compared to the NIR group. There were no IBA1 + cells in the PLX-treated IR group.

**Supplementary Fig. 3**. Activated BV2 cells impaired endothelial cell barrier function. BV2 cells were treated with or without LPS (1 µg/ml) for 72 h. Additional wells with BV2 culture medium with/without LPS but not BV2 served as controls. After 3 days, the culture medium was collected and applied to Transwell inserts and wells that were seeded with HUVEC. Diffusive solute flux assays were done after 24 h. LPS alone did not affect permeability of HUVEC. While culture medium from untreated BV2 strengthened HUVEC barrier function, culture medium from LPS-treated BV2 impaired HUVEC barrier function. *n* = 6 per group, where each dot represents an individual sample. * **p < 0.01, **** *p* < 0.0001, ns: *p* > 0.05 using one-way ANOVA.

**Supplementary Fig. 4**. Increased expression of *Tnf* and *C3* in Müller cells 3 days after IR. Expression was evaluated using the RiboTag method. No difference was found in expression of *Vegfa*. **p < 0.01, ns: *p* > 0.05 using Student’s *t* test.

**Supplementary Fig. 5**. PLX treatment reduced macrophage number, but not monocyte number in the spleen. Spleens were collected after 2 weeks of PLX treatment and analyzed by flow cytometry. There was a reduction in number of CD11b^+^CD45^+^Ly6G^−^CD11c^−^Ly6C^−^ macrophages. No difference was found in CD11b^+^CD45^+^Ly6G^−^CD11c^−^Ly6C^+^ monocyte number. *n* = 5 *p < 0.05, ns: *p* > 0.05 using Student’s *t* test.

## Data Availability

The data sets used and analyzed from the current study are available from the corresponding author upon reasonable request.

## Abbreviations

BRB Blood: Retinal barrier
iBRB: Inner blood-retinal barrier
oBRB: Outer blood-retinal barrier
BBB: Blood-brain barrier
CSF-1R: Colony-stimulating factor 1 receptor
PLX: PLX5622
IR: Ischemia reperfusion
NIR: Non-ischemia reperfusion
ERG: Electroretinogram
DR: Diabetic retinopathy
CRVO: Central retinal vein occlusion
