# Supplementary figures and images for: Suppression of inner blood-retinal barrier breakdown and pathogenic Müller glia activation in ischemia retinopathy by myeloid cell depletion
Source: J Neuroinflammation. 2024 Aug 24;21:210. doi: 10.1186/s12974-024-03190-9 (PMC11344463; doi:10.1186/s12974-024-03190-9)

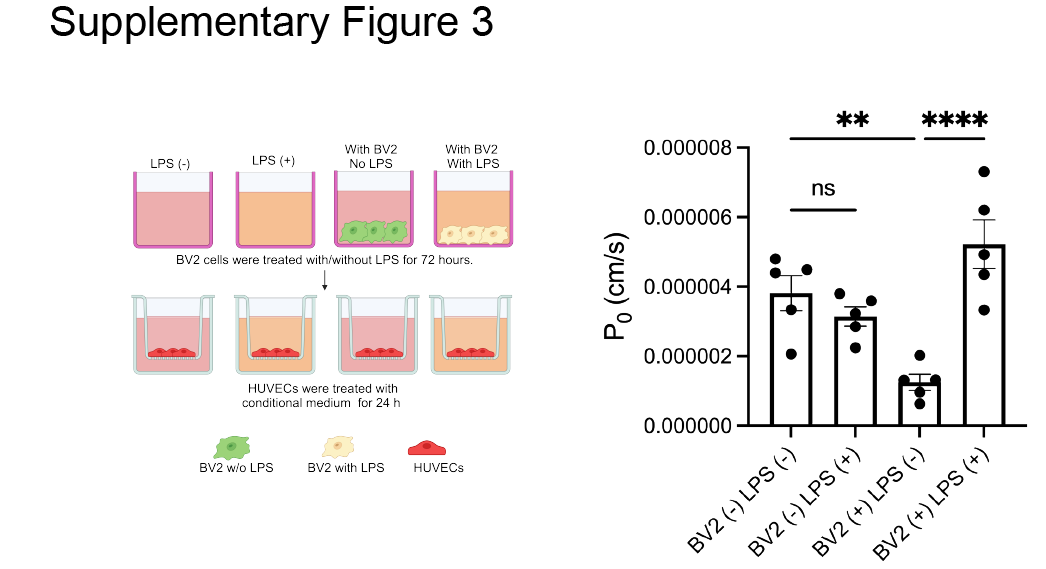

Supplement: Supplementary file 1 — Supplementary Fig. 1. Gating strategy for retinal flow cytometric analysis. Ly6G-positive cells were excluded prior to analysis of CD11b CD45 expression. [file 12974_2024_3190_MOESM1_ESM.tif]

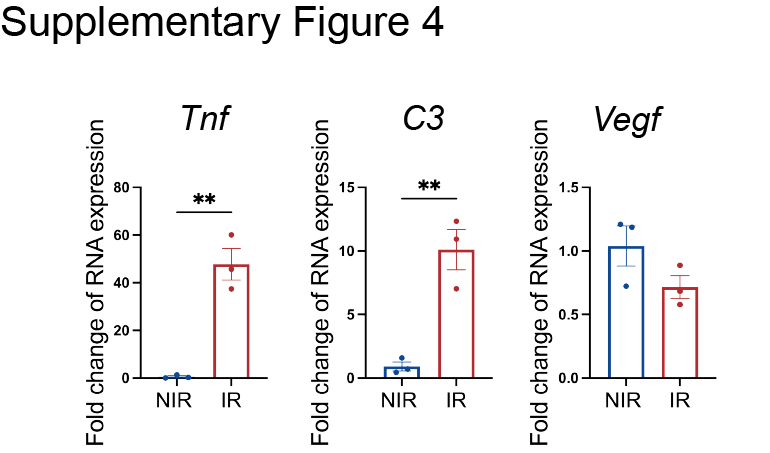

Supplement: Supplementary file 2 — Supplementary Fig. 2. Immunofluorescence staining of IBA1 in retinas collected after 3 days of IR. Myeloid cells were stained with IBA1 in red. There was a significant increase in IBA1 + cells in the IR Vehicle group compared to the NIR group. There were no IBA1 + cells in the PLX-treated IR group. [file 12974_2024_3190_MOESM2_ESM.tif]

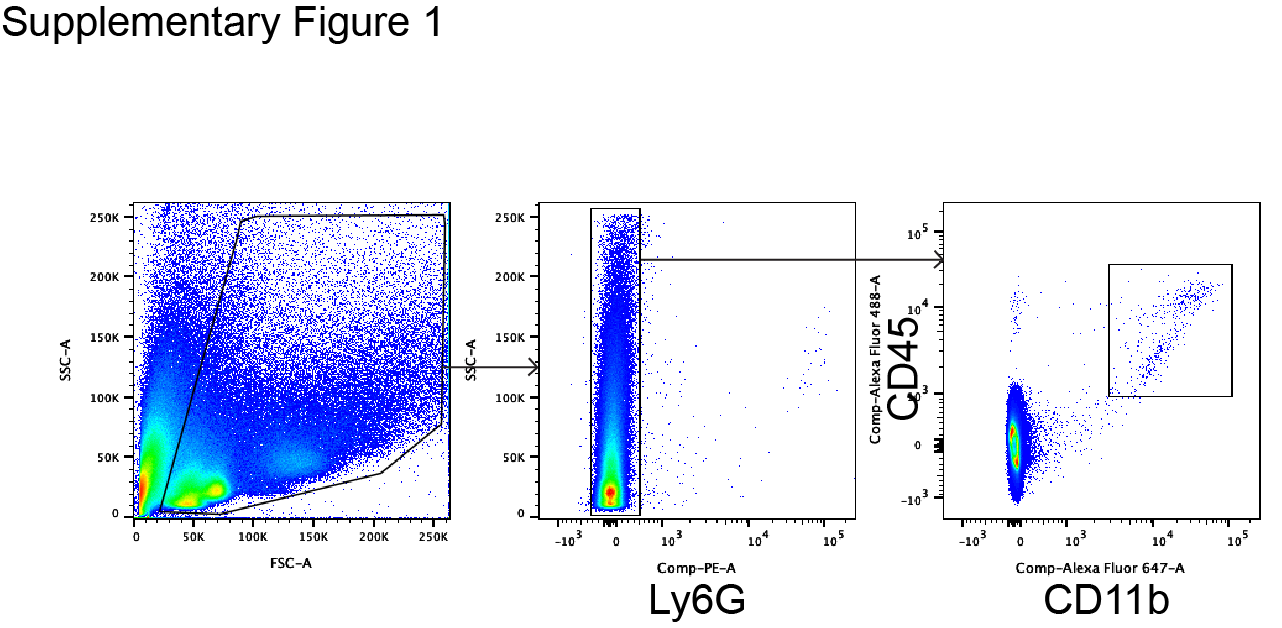

Supplement: Supplementary file 3 — Supplementary Fig. 3. Activated BV2 cells impaired endothelial cell barrier function. BV2 cells were treated with or without LPS (1 µg/ml) for 72 h. Additional wells with BV2 culture medium with/without LPS but not BV2 served as controls. After 3 days, the culture medium was collected and applied to Transwell inserts and wells that were seeded with HUVEC. Diffusive solute flux assays were done after 24 h. LPS alone did not affect permeability of HUVEC. While culture medium from untreated BV2 strengthened HUVEC barrier function, culture medium from LPS-treated BV2 impaired HUVEC barrier function. n = 6 per group, where each dot represents an individual sample. * **p < 0.01, **** p < 0.0001, ns: p > 0.05 using one-way ANOVA. [file 12974_2024_3190_MOESM3_ESM.tif]

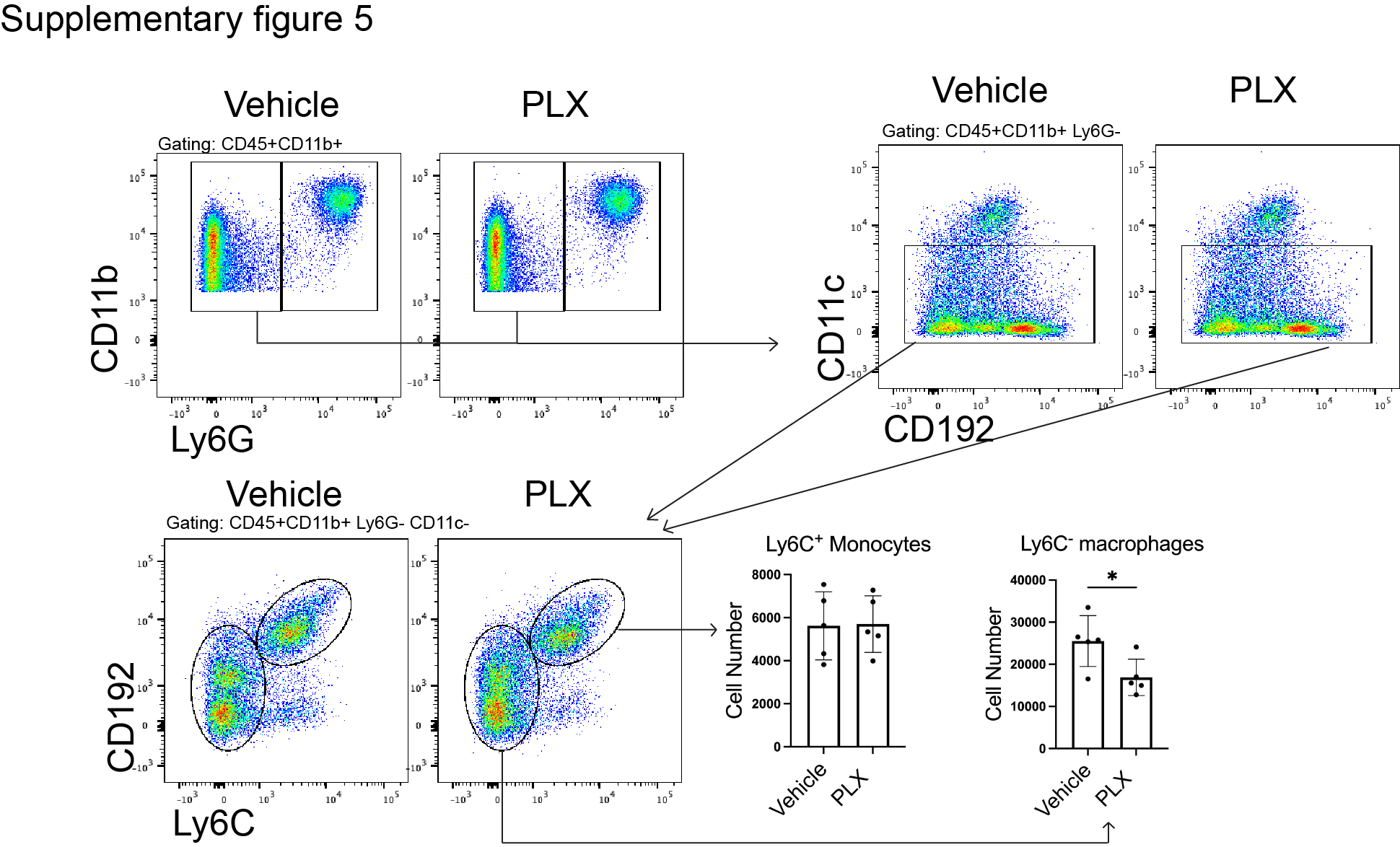

Supplement: Supplementary file 4 — Supplementary Fig. 4. Increased expression of Tnf and C3 in Müller cells 3 days after IR. Expression was evaluated using the RiboTag method. No difference was found in expression of Vegfa. **p < 0.01, ns: p > 0.05 using Student’s t test. [file 12974_2024_3190_MOESM4_ESM.tif]

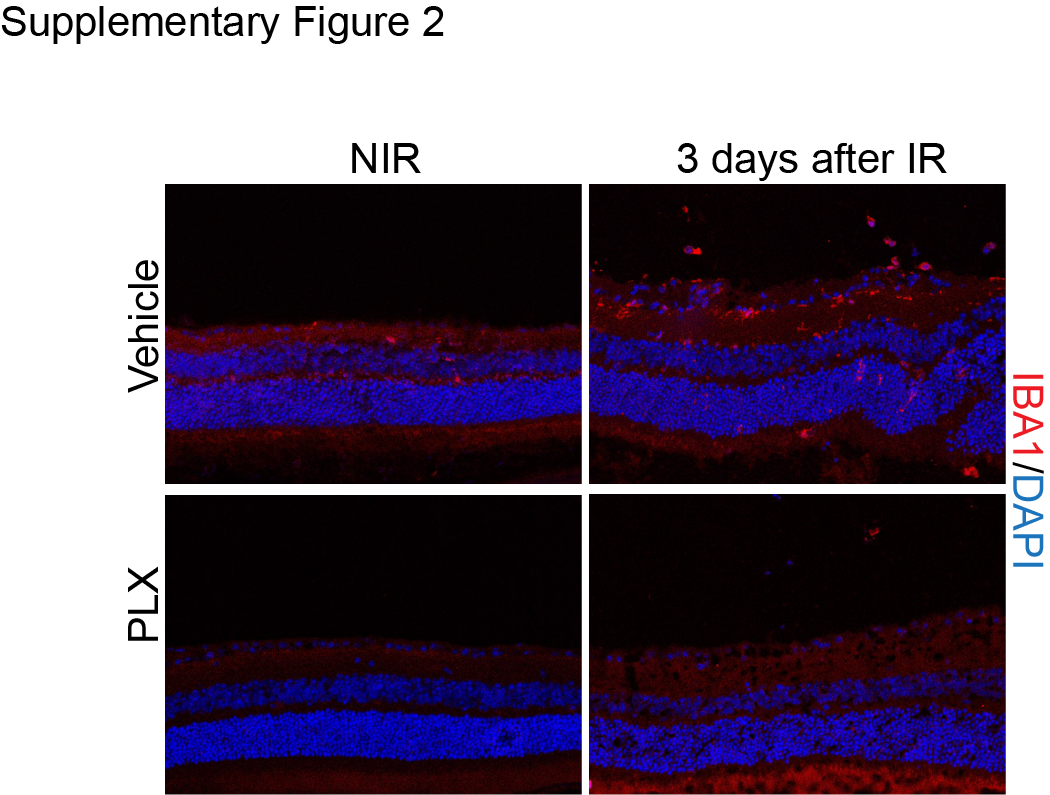

Supplement: Supplementary file 5 — Supplementary Fig. 5. PLX treatment reduced macrophage number, but not monocyte number in the spleen. Spleens were collected after 2 weeks of PLX treatment and analyzed by flow cytometry. There was a reduction in number of CD11b+CD45+Ly6G−CD11c−Ly6C− macrophages. No difference was found in CD11b+CD45+Ly6G−CD11c−Ly6C+ monocyte number. n = 5 *p < 0.05, ns: p > 0.05 using Student’s t test. [file 12974_2024_3190_MOESM5_ESM.tif]
